# Homœopathy

**Published:** 1881-01

**Authors:** Ad. Fellger

**Affiliations:** Phila.


### HOMŒOPATHY.

BY AD. FELLGER M.D. PHILA.

THERE are but two kinds of laws, the laws of God, and the laws of man. The laws of God or of nature, are absolute, infallible, and eternal, hence they can not be altered or corrected by man. The laws of man are changeable, fallible and mortal like man himself.* It is the duty of scientific men to discover

* The great difference between the laws of man and of nature seems to be understood by very few. We were once astonished to hear a professor, in a Homœopathic medical school, in arguing against the infallibility of our law, compare it to the "Laws of the Medes and Persians," which he said were once proclaimed to be unalterable; the natural inference being that the law of the similars was, like those laws, the fiat of a tyrant or master.—ED.

and investigate the laws of nature, for they form the fundamental basis of all science.

It is an undisputed fact that before Hahnemann's time, medicine was never guided in the exhibition of its drugs, by an eternal and infallible law. That grand, acute observer was the first to discover that law of nature which governed therapeutics and upon it he built the system of medicine which he called Homœopathy. This system will and must stand as long as the laws on which it is built remain,—that is, forever.

On the other hand the history of allopathic medicine shows that the medical profession in healing the sick, was either guided by experience without any principle; or by principles and speculative systems concocted in the erring brain of man; which theories have been invariably contradicted by experience. The falsity and uncertainty of men's theories will continue to be shown, by the practical test of experience, as long as they are not based on unchangeable natural laws. The brightest stars of the medical world, in all ages, have candidly confessed this fallacy; their lamentations about the uselessness of such methods, and their despair of ever coming to any certainty under such principles, should be sufficient testimony as to the utter worthlessness of their treatment; especially when this is compared with treatment based on Homœopathic laws. Therefore the true physician (the investigator of nature and its laws) has no choice as to the treatment he should select if he would be most successful in relieving the sufferings of mankind. His sound reason will prove to him that he must adopt and strictly adhere to the unchangeable principles discovered and laid down by Hahnemann, in other words, he must become a Homœopathic physician.

Truly has it been said, by Galen, that the best method of healing the sick is that which accomplishes it; *Cito, Tuto, Jucunde.* And we may, with equal truth, assert that no medical system except Homœopathy, does cure in this manner. Let us now see how amply Homœopathy *does* fulfil this desideratum of Galen.

Cito.—It is well known to all Homœopathic physicians how wonderfully quick the manifold symptoms, incident to diseased conditions, are alleviated, also that even the most violent affections, though of long duration, or worse, aggravated by previous bad treatment, are promptly mitigated or completely cured, by the application of the law of the similars. These results we must attribute to the correct principles of Homœopathy; results which the old school can not produce even with its narcotica and anæsthetica, which drugs bring but temporary relief and seldom cure.

In the overwhelming majority of cases treated thus, *i. e.* palliatively, the original disease returns again and again, with augmented intensity, until the patient's constitution is totally wrecked, and its restoration to health is rendered well nigh an impossibility. The number of such cases is legion. It is my intention to narrate several marked cases of this kind in a subsequent paper.

Tuto.—Dr. Wolf, of Berlin writes, as early as 1858: "The Homœopathic physician is not only able to cure positively those diseases which have been shown up to the present time to be curable, but he can also eradicate from the human system the tendency to new morbid affections, as well as the causes of life-long infirmity, thereby preventing the continuance of disease from generation to generation." Whereas the allopathic school, with its massive doses and its so-called specific remedies suppresses disease, thereby engendering new and worse diseased conditions, which eventually leads to confirmed ill health or even to death. What Homœopathic physician is not cognizant of the injurious effects of mercury, quinia, the narcotica, drastica, etc., etc.? The countless detrimental effects consequent upon allopathic medication are spared mankind by the Homœopathic method of cure, for Homœopathy never suppresses an ailment; or transforms it into a chronic diseased condition, one injurious to health or even dangerous to life.

Jucunde.—In this particular, Homœopathy has surely verified the most sanguine expectations that could possibly be entertained of any art of healing. Not only this, but it has completely done away with the whole allopathic* apparatus of torture, such as the moxa, the seton, blistering, cauterizing (even to the bone) etc; it has done more yet, for it has caused those nauseous and disgusting drugs recently in use, to be thrown aside. Such diseases as tumors, fistulæ, strabismus, condylomata, etc., for the cure of which the old school has no other means than the knife or the cautery, are healed by Homœopathic remedies, when indicated by the eternal law of the similars. Not only does Homœopathy cure the above mentioned diseases, but it also improves the constitution of the patient, and hence prolongs life. The operative procedures of the old school never eradicate the real *internal* diseased condition (of which tumors, etc., etc., are the mere manifestation), on the contrary it is brought into a more rapid decline and, not seldom, to a speedy death. We can proudly claim that the influence of Homœopathy on the old school has been so great that it has forced them to abandon, in great part, those barbarous methods which were in vogue before the days of Hahnemann. Had Homœopathy accomplished nothing more than this, and could it claim no more favorable results in the treatment of the sick than allopathy, even then it would still rank pre-eminent in medicine. What man of feeling would gainsay this when he remembers the unspeakable misery and unlimited suffering, which have become things of the past since the advent of Homœopathy.

The disciples of Hahnemann are counted by thousands, their followers by millions, and are spread broadcast over all lands;

* To illustrate how little the allopaths have sought to fulfil this "*Jucunde* treatment" we instance—*e pluribus unum*—the following: Magendie, the physiologist in discussing etherization before the French Academy of Sciences, declared that "pain always has its usefulness," also declared it to be "a trivial matter to suffer and a discovery, whose object was solely to prevent pain was of a slight interest only."—(*Gazette Medical de Paris*, Feb. 6th, 1847, p. 112). Simpson says, "Hildanus, the patriarch of German surgery, amputated the limbs of his patients with red-hot knives, in order that he might divide the flesh and sear up the vessels at one and the same time." Not much "*Jucunde*" in this practice?—ED.

this in spite of all the efforts of their opponents. The power of the truth and the success of Homœopathy were so convincing that many of its bitterest and most learned enemies became its staunch defenders, as soon as they were willing to undertake a fair trial of its law. He who knows the fate of Harvey, Auenbrugger, Mesmer, Reichenback and others, and who remembers that Velpeau, in 1835, as president of the Medical Society in Paris, rejected all communications concerning anæsthetics as chimerical, with the remark, that such fantastical ideas were not worthy of the consideration of scientifically educated physicians.* I repeat, he who knows all this will cease to wonder that Hahnemann also, when he first promulgated his immortal theory of Similia, experienced the same fate. All, even his best friends, although honoring his talents and immense learning, turned against him, and derided his discovery.

The law of the Similia, the single dose, the minute dose, the habit of allowing ample time between doses for them to act to their fullest extent, the proving of drugs on the healthy—all these, seeming innovations, contrasted so strongly with the practice of medicine then in vogue, that the scientific men of Hahnemann's time found it a very easy task to cover his great discovery with ridicule.

The whole medical world of that day sat in solemn judgment on Hahnemann's theory, *without* ever inquiring into the facts. Instead of following the experimental method of Galileo they adopted the logical method of Aristotle, and so passed sentence upon his discovery without adequate practical knowledge of it. All knowledge and erudition were marshalled in defense of the old doctrines in medicine and for the annihilation of the new.

* In speaking of Ambrose Pare's discovery of the ligature, Cooper says: "Almost one hundred years after Pare, a button of vitriol was employed, in the Hotel Dieu, at Paris, for the stoppage of hemorrhage. . . . So difficult was it to eradicate the *blind* attachment shown for the ancients, that Baronius, a professor at Cremona, publicly declared, in 1609, that he would rather err with Galen than follow the advice of any other physician!" ("Cooper's Dictionary of Practical Surgery," 7th ed., p. 46).—ED.

Thus a very learned physician* of Hamburg wrote a book against the "Organon," exceeding it in volume five fold, in which nearly all the ancient classical writers of Greece and Rome are cited. Having read this book, we involuntarily arrive at the same conclusion, as after reading Tycho de Brahe's works and polemics against the Copernican system; namely, that no doubt not only an immense amount of learning is necessary to compile such works, but also that all the learning of the world does not suffice to cast eternal truths into doubt, or to philosophize errors into their place; and furthermore, that it is an impossibility for one not so well versed in the classics to say so many silly and absurd things.

Only slowly and by degrees did Hahnemann, by the success of his method of treatment, prove to the world the truth of his theory and thereby gain proselytes. As the observations of Hahnemann were verified daily in the practice of his followers, it was but natural that Homœopathy should rise to its present eminence, in spite of all opposition, even of governments egged on by influential lay and medical men. If we adhere, with the utmost strictness, to the principles bequeathed us by Hahnemann in his "Organon," the time will not be far distant when Homœopathy will reign supreme everywhere as the benefactor of suffering mankind.

It is true that many deceive themselves with regard to the amount of unremitting mental labor which is necessary in order to be an able Homœopathic physician. For one must toil continuously, as long as life lasts, to overcome the difficulties and problems presenting themselves in endless succession, as Homœopathy advances. Routine *can* never be acquired by the *true* follower of Hahnemann, as every new case must be individualized and treated strictly according to the law of similia. The

* As another instance of this tendency to erroneous opinions on the part of those supposed to be learned in any branch, and also to show how one not so learned (professionally) may form a more correct opinion, we would mention the following. In 1856 an engineer wrote a long dissertation, in the *Edinburgh Review*, in which he scientifically proved "the absurdity" of the Suez Canal. Yet that "absurdity" was afterward built by one who was not a professional engineer.—Ed.

closer the physician adheres to this law, and the more conscientiously he follows its precepts, the more he will be rewarded by success; and the more will he be admonished of the necessity of continued study. With such experience as may be at my disposal, I shall endeavor, on a future occasion, to illustrate some of the great difficulties in the path of every practical physician.

Whilst we adhere rigidly to the principles of the "Organon," we must not fail to appropriate all new discoveries which may extend the application of Homœopathy, be they discoveries in hygiene, dietetics, or medicine proper. We must also acknowledge our indebtedness to the scientists who have, since the origin of Homœopathy, made many discoveries tending to prove the correctness of its principles.

I will offer proof, at another time, that Homœopathy, with its principles, in the treatment of the sick, occupies a higher plain than allopathy. Judging from the proofs which science is rapidly accumulating for us, we may say positively, that in the course of time allopathy will be forced to admit the correctness of the principles of Homœopathy. I quote the closing words of Prof. Jaeger in his report on Neuralanalysis to the convention of physicists at Dantzig.

"The graduated increase in the physiological action of drugs as we raise their potency, being shown by Neuralanalysis, elevates Homœopathy at once to the position of an exact physiologically proven science and places it positively on a level with allopathy. In consideration of this fact, it will in the future be an impossibility for the old school to combat systematically the doctrines of Hahnemann and, by the power of Neuralanalysis, Homœopathy will take its place on the roster of the Universities."

